# Turning the tide in HIV through health communication research

**DOI:** 10.1002/jia2.25955

**Published:** 2022-06-23

**Authors:** Collene Lawhorn, Michael J. Stirratt, Dianne M. Rausch

**Affiliations:** ^1^ Division of AIDS Research, National Institute of Mental Health Rockville Maryland USA

1

Effective communication is critical to health promotion. The growth of digital communication offers important opportunities for global information exchange and social media influences to enhance HIV health promotion [1]. At the same time, this dynamic information ecosystem contributes to the amplified proliferation of misinformation, disinformation and poorly delivered communication. During the COVID‐19 pandemic, strategic messaging from trusted sources was important for advancing vaccine acceptance and combating an upsurge in vaccine misinformation worldwide [2, 3]. The importance of health communication has been similarly apparent throughout the HIV pandemic––from the longstanding challenges of AIDS denialism and HIV stigma, through current efforts to advance the contemporary message that Undetectable = Untransmittable (U = U) [4]. The future design and delivery of high‐impact HIV health communication will require combination approaches and models that integrate discovery, translation and implementation science. Which tools, principles and practices from communication science, behavioural and social science and HIV research could inform our efforts?

Communication science focuses on how information is created, shared, received, understood and responded, drawing from multiple disciplines, including media studies, marketing, psychology and political science. Communication science invites close attention to the source, channel and receiver when framing health‐related messages, and provides specific methods and novel tools that may benefit HIV research and practice. Communication methods that involve the rapid creation of large, locally or nationally representative research panels through internet‐based recruitment invite perspectives from key populations who might otherwise go unreached [5]. Audience segmentation approaches that use response patterns can help identify categories of individuals who may be open, persuadable or resistant to various HIV‐related health messages [6]. Studies of digital HIV interventions that use marketing tools, such as A/B testing, a method that enables contrasting messages to be tested to determine which performs better, may elucidate which messages foster greater reach and engagement in real time.

Integrating health communication tools with behavioural and social science theories [7, 8, 9] may improve learning, motivation and multilevel impact in HIV. One example is behavioural economics, which combines tenets from psychology and economics to understand influences on human decision making and behaviour and leverages cognitive processes to “nudge” people to make healthy choices and adopt healthy behaviours. The application of behavioural economics to health communications research can open new opportunities. A recent behavioural economics “mega study” sought to promote influenza vaccination completion by concurrently testing more than 19 variations in text message content and frequency among more than 47,000 patients in two large healthcare systems [10]. The communication nudges that were associated with the greatest increases in vaccine receipt offered two reminder scripts that indicated “a vaccine has been reserved for you” [10]. Similarly large, communication and behavioural economic‐based studies could also help drive appointment completion for HIV treatment or pre‐exposure prophylaxis (PrEP).

Creating effective health communications in HIV requires building on the long tradition of community‐centred advocacy and efforts in HIV. Community‐informed approaches helped catalyse sex‐positive messaging for condom promotion. The Denver Principles, articulated in 1983 by a group of gay men living with HIV, pioneered replacement of stigmatizing language (previously, “AIDS victims”) with empowering and destigmatizing people‐first language (“people living with HIV”) [11, 12]. Currently, advocates have built on the science of HIV treatment as prevention to develop campaigns like U = U, which have been found to be meaningful and destigmatizing by people living with HIV and their partners. A growing evidence base demonstrates that using a community‐informed messaging campaign like U = U whose foundation is strong, community‐centred and advocacy‐driven, helps mitigate stigma and positively impacts HIV knowledge, testing and viral suppression [13, 14]. These examples tell us that HIV health communication can have meaningful traction when community input serves as its foundation.

How can communication science, behavioural and social science, and community‐informed approaches in HIV be integrated into a whole that is primed for impact? Research to further advance the global uptake and impact of U = U offers one example. Communication science could further current efforts and inform tailored interventions by using large panels and audience segmentation approaches to better understand those who accept, resist or may be persuaded by U = U messaging. Behavioural economic principles could help us to consider and test the optimal message framing, timing and delivery of the U = U message.

Beyond U = U, future research must also include (1) understanding influential sources in the contemporary communication landscape and the impact of HIV health messages on prevention, treatment and cure efforts; (2) optimizing HIV‐related interpersonal communication that drives inclusivity, champions respect for consumers’ diverse health needs, improves health literacy and eliminates stigma; and (3) strengthening HIV information coordination and dissemination for broad audiences (i.e. scientific, consumer, community and policy) while striving to combat misinformation and disinformation. To inform our efforts and help us develop a model for a future communication research agenda, we have articulated a model (Figure 1) that identifies a core communication research agenda through the interplay of discovery, translational and implementation science. The model illustrates a continuous feedback loop among three key areas of communication research––where discovery science informs translation, translational science informs implementation and implementation science contributes to discovery. Building on the best traditions and lessons of HIV health communication to date, the model also illustrates how the most effective health communications will not be crafted for communities, but with communities, centring community perspectives and context for greater reach and impact [7, 15].

**Figure 1:**
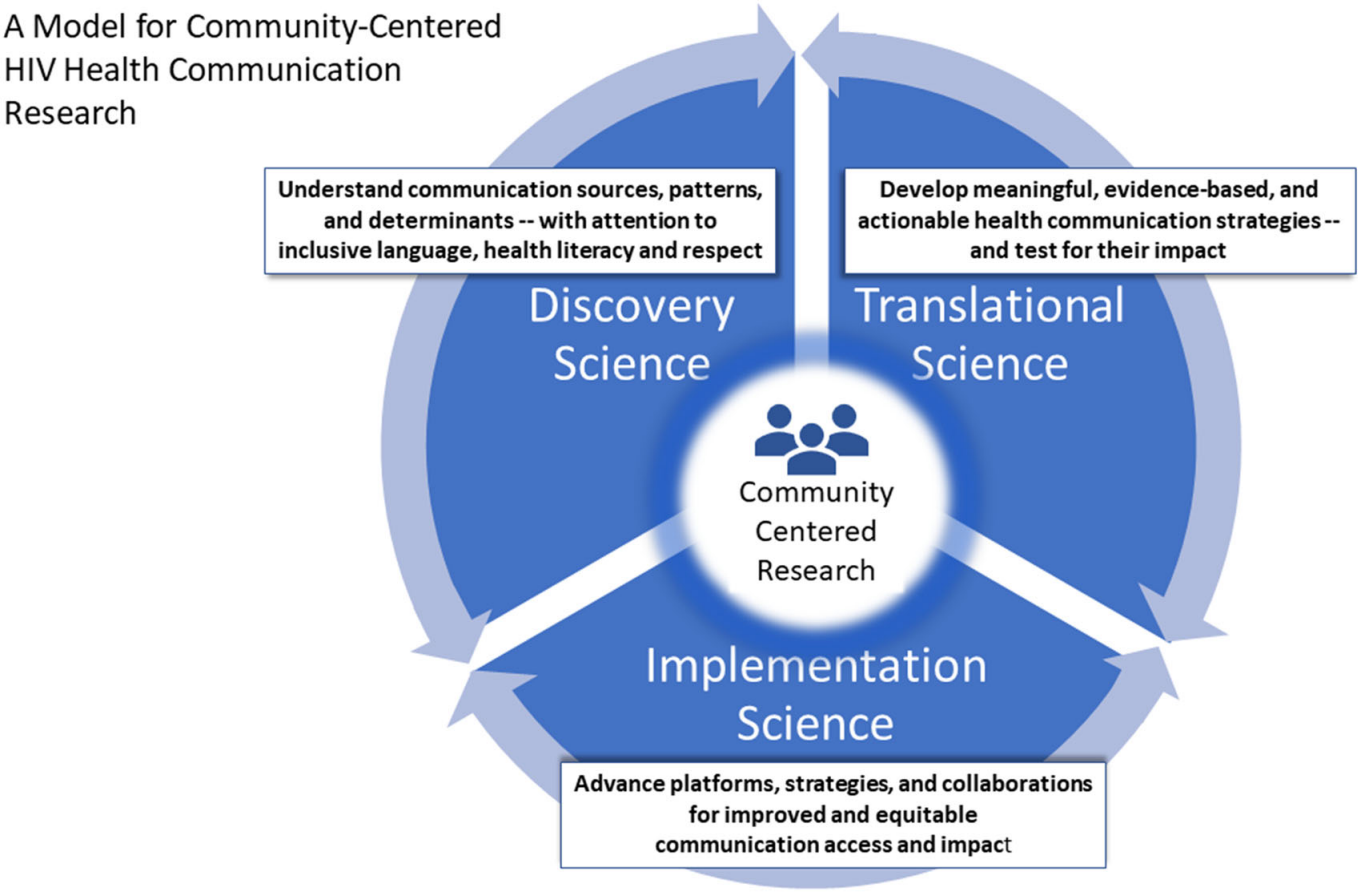
This model illustrates a continuous feedback loop and interplay among key areas of health communication research that includes discovery science, translational science and implementation science.

The rapidly diversifying set of HIV prevention and therapeutic options demonstrates the critical need and utility of a community‐centred health communication research model. For example, the adoption of novel regimens like long‐acting injectables alongside daily oral antiretroviral therapy and PrEP might require insights from basic research to identify effective and preferred communication channels among key populations; translational research to strengthen informed decision making around choice and adoption of approved HIV products; and implementation strategies to disseminate approaches for strengthening community‐centred knowledge.

The evolving information ecosystem will require fresh, strategic approaches with input from those with lived experience, scientists, community partners, advocates, providers, technologists and policymakers. To accelerate global efforts to end HIV, all of these contributors need to refocus our research efforts on health communication science. Words matter, and they can make a difference.

## COMPETING INTERESTS

The authors have no competing interests.

## AUTHORS’ CONTRIBUTIONS

CL, MJS and DMR equally developed and wrote the initial and revised versions of the manuscript. CL, MJS and DMR have read and approved the final manuscript.

## DISCLAIMER

The opinions expressed in this viewpoint do not necessarily represent the views of the National Institutes of Health, the Department of Health and Human Services or the United States Government.

## References

[1] Maloney KM , Bratcher A , Wilkerson R , Sullivan PS . Electronic and other new media technology interventions for HIV care and prevention: a systematic review. J Int AIDS Soc. 2020;23(1):e25439. 31909896 10.1002/jia2.25439 PMC6945883

[2] Latkin CA , Dayton L , Yi G , Konstantopoulos A , Boodram B . Trust in a COVID‐19 vaccine in the U.S.: a social‐ecological perspective. Soc Sci Med. 2021;270:113684. 33485008 10.1016/j.socscimed.2021.113684 PMC7834519

[3] Puri N , Coomes EA , Haghbayan H , Gunaratne K . Social media and vaccine hesitancy: new updates for the era of COVID‐19 and globalized infectious diseases. Hum Vaccin Immunother. 2020;16(11):2586–93. 32693678 10.1080/21645515.2020.1780846 PMC7733887

[4] Kalichman SC , Eaton L , Cherry C . “There is no proof that HIV causes AIDS”: AIDS denialism beliefs among people living with HIV/AIDS. J Behav Med. 2010;33(6):432–40. 20571892 10.1007/s10865-010-9275-7 PMC3015095

[5] Callegaro M , Allum N , Sturgis P . Online panel research: history, concepts, applications and a look at the future. In Callegaro M , Baker RP , Bethlehem J , Göritz AS , Krosnick JA , Lavrakas PJ , editors. Online panel research: a data quality perspective. 2014: 1–22.

[6] Gomez A , Loar R , Kramer AE , Garnett GP . Reaching and targeting more effectively: the application of market segmentation to improve HIV prevention programmes. J Int AIDS Soc. 2019;22(Suppl 4):e25318. 31328397 10.1002/jia2.25318 PMC6643068

[7] Airhihenbuwa CO, Obregon R . A critical assessment of theories/models used in health communication for HIV/AIDS. J Health Commun. 2000;5(Suppl):5–15. 11010357 10.1080/10810730050019528

[8] Babalola S , Van Lith LM , Mallalieu EC , Packman ZR , Myers E , Ahanda KS , et al. A framework for health communication across the HIV treatment continuum. J Acquir Immune Defic Syndr. 2017;74(Suppl 1):S5–14. 27930606 10.1097/QAI.0000000000001206 PMC5147045

[9] Rausch DM , Grossman CI , Erbelding EJ . Integrating behavioral and biomedical research in HIV interventions: challenges and opportunities. J Acquir Immune Defic Syndr. 2013;63(Suppl 1):S6–11. 23673890 10.1097/QAI.0b013e318292153b

[10] Milkman KL , Patel MS , Gandhi L . A megastudy of text‐based nudges encouraging patients to get vaccinated at an upcoming doctor's appointment. Proc Natl Acad Sci USA. 118(20):e2101165118. 10.1073/pnas.2101165118 PMC8157982 33926993

[11] Dilmitis S , Edwards O , Hull B , Margolese S , Mason N , Namiba A , et al. Language, identity and HIV: why do we keep talking about the responsible and responsive use of language? Language matters. J Int AIDS Soc. 2012;15(S2):17990.

[12] Advisory Committee of the People with AIDS . The Denver Principles. 1983.

[13] Bor J , Fischer C , Modi M . Changing knowledge and attitudes towards HIV treatment‐as‐prevention and “Undetectable = Untransmittable”: a systematic review. AIDS Behav. 2021;25(12):4209–24. 34036459 10.1007/s10461-021-03296-8 PMC8147591

[14] Smith P , Buttenheim A , Schmucker L , Bekker LG , Thirumurthy H , Davey DLJ . Undetectable = Untransmittable (U = U) messaging increases uptake of HIV testing among men: results from a pilot cluster randomized trial. AIDS Behav. 2021;25(10):3128–36. 34057659 10.1007/s10461-021-03284-y PMC8165342

[15] Peinado S , Treiman K , Uhrig JD , Taylor JC , Stryker JE . Effectively communicating about HIV and other health disparities: findings from a literature review and future directions. Front Commun (Lausanne). 2020;5, 10.3389/fcomm.2020.539174 PMC7884094 33594338

